# Outcomes of Combined Liver and Pancreas Transplantation: A Review of the SRTR National Database and a Report of the Largest Single Center Series

**DOI:** 10.3389/fmed.2020.542905

**Published:** 2020-10-19

**Authors:** Cheukfai Li, Wei Zhang, Qiang Zhao, Maodong Ye, Weiqiang Ju, Linwei Wu, Yi Ma, Anbin Hu, Guodong Wang, Xiaofeng Zhu, Zhiyong Guo, Dongping Wang, Xiaoshun He

**Affiliations:** ^1^Organ Transplant Center, The First Affiliated Hospital, Sun Yat-sen University, Guangzhou, China; ^2^Department of Breast Cancer, Cancer Center, Guangdong Provincial People's Hospital and Guangdong Academy of Medical Science, Guangzhou, China; ^3^Department of Medical Ultrasonics, Third Affiliated Hospital of Sun Yat-sen University, Guangzhou, China

**Keywords:** scientific registry of transplant recipients (SRTR), the First Affiliated Hospital (FAH), Sun Yat-sen University, simplified multivisceral transplantation (SMT), type 2 diabetes mellitus (T2DM), end-stage liver disease (ESLD)

## Abstract

**Purposes:** This study was intended to summarize the characteristics and clinical outcome of Liver and Pancreas (LPTx) recipients in the Scientific Registry of Transplant Recipients (SRTR) database vs. the largest series from the First Affiliated Hospital (FAH), Sun Yat-sen University.

**Methods:** The clinical data of 23 patients who underwent LPTx from 2000 to 2016 in the United States and 31 patients who underwent modified LPTx procedure (known as simplified multivisceral transplantation [SMT]) from 2008 to 2017 in our center were reviewed. The indications, surgical techniques, patient and graft survival, and complications were compared between the two groups.

**Results:** All recipients in the FAH group were diagnosed with type 2 diabetes mellitus, while 10 of 23 recipients were diagnosed with type 1 diabetes mellitus in the SRTR group. The 1-, 3-, and 5-year cumulative patient survival rates were 81, 74, and 74% in the FAH group, respectively, and 51, 47, and 37% in the SRTR group, respectively (*P* = 0.023). No diabetes was observed during follow-up in the FAH group, while the diabetes recurrence rate was 22.2% in the SRTR group (*P* = 0.03).

**Conclusion:** With multiple techniques modified and indications changed, the SMT procedure yielded a preferable outcome compared to that of the traditional LPTx procedure in records of SRTR. SMT has become a treatment option for patients with end-stage liver disease and concurrent diabetes.

## Introduction

Combined liver and pancreas transplantation (LPTx) is not a common procedure in the history of transplantation. In the 1990s, reports of LPTx were often included in abdominal multivisceral transplantation as a treatment for unresectable abdominal cancer (1). The operation course was generally uneventful, but most patients died of tumor recurrence, which significantly restricted development of this technique.

Nevertheless, increased insulin resistance and impaired glucose metabolism are common among patients with end-stage liver diseases (ESLD) (1, 2). It has been documented in many studies that the outcomes, especially the long-term survival rate of liver transplant recipients with pretransplant diabetes, is inferior to those without diabetes (3–6). Orthotopic liver transplantation (OLT) alone cannot reverse the impaired glucose metabolism in patients with ESLD and concurrent insulin-dependent diabetes, which might even be aggravated by the use of immunosuppressants post-transplantation (7). Of note, persistent diabetes has posed great challenges during post-transplant management and has negatively affected the outcomes of OLT (8). LPTx has been proven to be a possible treatment for patients with ESLD and concurrent type 1 diabetes mellitus (T1DM) (9, 10), while its use in ESLD and concurrent insulin-dependent type 2 diabetes mellitus (T2DM) remains a matter of debate. We reported a modified LPTx procedure, called simplified multivisceral transplantation (SMT), for the treatment of patients with ESLD and concurrent T2DM (11).

To evaluate the safety and efficacy of LPTx for the treatment of patients with ESLD and concurrent diabetes, we reviewed the indications, patient and graft survival, and post-transplant complications of all LPTx recipients in the United States from the Scientific Registry of Transplant Recipients (SRTR) database and the largest SMT cohort from a single Chinese center.

## Patient and Method

### Patients

This study was performed in accordance with the Declaration of Helsinki and was approved by the institutional review board at Sun Yat-sen University. Due to the retrospective and observational nature of the research, the need for written informed consent was waived.

This study was based on an analysis of data from the SRTR data system, which includes data on all donors, waiting list candidates, and transplant recipients in the United States, submitted by the members of the Organ Procurement and Transplantation Network (OPTN). The Health Resources and Services Administration (HRSA), U.S. Department of Health and Human Services, oversees the activities of the OPTN and SRTR contractors.

The study population consisted of two cohorts of patients. Seventy-two cases of LPTx from the SRTR national database between 1988 and 2016 were recorded (SRTR group). However, some of the data in the late 1980s and 1990s were missing. In order to appropriately compare a similar period of cohorts, Twenty-three cases of LPTx from SRTR between 2000 and 2016 were selected for further analysis. Thirty-one cases of SMT were conducted between 2008 and 2017 in the Organ Transplantation Center, the First Affiliated Hospital of Sun Yat-sen University (FAH group). Recipients with a previous transplant or age <18 were excluded.

Patient characteristics were recorded, including gender, age, diagnosis, body mass index (BMI), model of end-stage liver disease (MELD) score, and hospital stays. Donor characteristics were also recorded, including gender, age, BMI, donation category, and cold ischemia time. Patient and graft survival and post-transplant complications were documented.

### Surgical Procedure

Our previous report (11) described in detail the surgical procedure of SMT in the FAH group. The procedure of organ donation and procurement strictly followed the Chinese guideline of organ donation (12). Briefly, procurement of organ cluster was carried out according to standard surgical technique. The diseased liver was excised with the recipient's pancreas remaining. The “en-bloc” liver and pancreas graft were implanted *in situ* using a piggyback procedure. For artery reconstruction, the donor's superior mesenteric artery and celiac trunk artery were anastomosed to the donor's internal and external iliac artery, during the back-table procedure. During implantation, the opening of the iliac artery was anastomosed to the recipient's common hepatic artery. After this reconstruction, the artery blood flow was supplied through a Y-shape aortic bypass to the liver-pancreas-duodenum graft. The recipient's portal vein was anastomosed to the posterior wall of the graft portal vein in an end-to-side fashion. After the vascular anastomosis, the clamps of the artery and vein were released simultaneously. For digestive tract reconstruction, the graft's proximal and distal duodenum were closed as a C-loop. In the first six cases performed in FAH, recipients' jejunum was anastomosed end-to-side to donor duodenum using the Roux-en-Y technique. In the following 25 cases, side-to-side anastomosis of the donor to recipient duodenojejunal anastomosis was performed. The duodenal depression tube was maintained for post-operation 4 weeks. In the SRTR database, information concerning the surgical techniques was included, such as implantation technique, venous vascular management, and exocrine drainage management.

### Immunosuppressive Protocol

In the FAH group, induction therapy was performed by administration of a dose of 20 mg basiliximab (anti-IL-2 receptor antibody; Simulect, Novartis Pharma AG, Basel, Switzerland) intraoperatively and on post-transplant day 4. A dose of 500 mg methylprednisolone was given intraoperatively. Tacrolimus and mycophenolate mofetil (MMF) were given 4 days after the operation. The initial dose of tacrolimus was 0.04 mg/kg/d, and the target trough level was 8–10 ng/ml within the first 3 months, and 6–8 ng/ml thereafter. A dose of 500–750 mg MMF was given twice a day. Induction therapy and maintenance drugs were documented in immunosuppressive records in the SRTR, whereas the dose of each immunosuppressant was unknown.

### Statistical Analysis

Data were expressed as median (interquartile range) or mean ± standard deviation as appropriate. Categorical variables were compared using chi-square test or Fisher's exact test, while continuous variables were compared using Mann–Whitney *U*-test or *t*-test. Survival analysis was conducted using the Kaplan-Meier method, and two groups were compared by the log-rank test. *P*-values were two-tailed and were statistically significant if <0.05. All statistical analyses were conducted by using Statistical Product and Service Solutions 20.0 (SPSS, Inc., Chicago, IL).

## Result

### Clinical Characteristics

The baseline characteristics in the SRTR and FAH groups are summarized in Table 1. There were 14 male recipients (60.9%) in the SRTR group and 30 male recipients (96.8%) in the FAH group. The mean age of recipients was 43.2 ± 14.6 (range: 23–65) years in the SRTR group and 56.1 ± 9.8 (range: 33–73) years in the FAH group. The mean MELD score of recipients was 22.6 ± 7.6 (6–40) in the SRTR group and 19.0 ± 12.3 (6–49) in the FAH group. Median follow-up time was 49.4 months (interquartile range: 4.9–88.3; range: 0–119.6) in the SRTR group and 13.0 months (interquartile range: 2–28; range: 0–78) in the FAH group (*P* = 0.11). All the donation categories in the SRTR group were donation after brain death (DBD), while only 41.9% donors were DBD donors in the FAH group. Nevertheless, no significant difference was observed in BMI of recipients, and gender, age, cold ischemia time, and BMI of donors between the two groups (all *P* > 0.05).

**Table 1:** Summary of patient and donor characteristics.

	**FAH (*n =* 31)**	**SRTR (*n =* 23)**	***P***
**RECIPIENT CHARACTERISTICS**			
Gender (male)	30/31	14/23	0.003
Age (years)	56.1 ± 9.8 (33–73)	43.2 ± 14.6 (23–65)	0.001
BMI (kg/m^2^)	23.7 ± 3.6 (18.8–32.1)	23.2 ± 5.6 (16.24–34.5)	0.70
MELD score	19.0 ± 12.3 (6–49)	22.6 ± 7.6 (6–40)	0.047
Follow up (months)	13.0 (2, 28) (0–78)	49.4 (4.9, 88.3) (0–119.6)	0.11
Hospital stays (days)	37(26, 47) (10–138)	14(11.5, 24.75) (0–148)	<0.001
**DIAGNOSIS**			
Pancreas diagnosis			<0.001
Diabetes mellitus-type I	0/31	10/23	
Diabetes mellitus-type II	31/31	5/23	
Other (pancreatic cancer, cystic fibrosis, etc.)	0/31	8/23	
Liver diagnosis			<0.001
HCC	18/31	2/23	
Cirrhosis	11/31	10/23	
Cholangiocarcinoma	1/31	0/23	
Malignancy	0/31	1/23	
Other	1/31	10/23	
Hepatitis virus			
HBV N/P	1/30	21/1	<0.001
HCV N/P	31/0	20/2	0.168
**DONOR CHARACTERISTIC**			
Gender (male)	24/31	15/23	0.08
Age (years)	29.0 ± 11.8 (10–56)	24.7 ± 8.2 (8–41)	0.13
BMI (kg/m^2^)	21.4 ± 2.8 (17.0–26.7)	22.6 ± 4.4 (13.8–35.4)	0.36
Donation category			<0.001
DBD	13	23	
DCD	17	0	
DBCD	1	0	
Cold ischemia time (hour)	7.7 ± 1.3 (6.0–11.0)	7.9 ± 2.6 (4.5–15.3)	0.65

*FAH, the First Affiliated Hospital, Sun Yat-sen University; SRTR, Scientific Registry of Transplant Recipients; BMI, body mass index; MELD, model for end-stage liver disease; HCC, hepatocellular carcinoma; HBV, hepatitis B virus; HCV, hepatitis C virus; DBD, donation after brain death; DCD, donation after cardiac death. Estimates of variance are standard deviations*.

### Indications

It is noteworthy that the primary diseases were different between the two groups. There were various indications in the SRTR group, including hepatocellular carcinoma (HCC), cirrhosis, cholangiocarcinoma, and cystic fibrosis (CF), while the main indications in the FAH group were HCC and cirrhosis. In the FAH group, 30 of 31 recipients were HBV infected, while the infection rates of HBV and HCV were both significantly lower in the SRTR group. The diabetes mellitus types of recipients were quite different as well. There were 10 recipients diagnosed with T1DM and five with T2DM in the SRTR group, while all 31 recipients in FAH group were diagnosed with T2DM. The primary diagnosis of pancreas in SRTR group also included pancreatic cancer, pancreatectomy, and CF in the other eight recipients.

### Surgical Technique

During recipient operation, reconstructions of venous outflow, vascular inflow, and exocrine drainage were performed to ensure graft function. The operation techniques used were different between the two groups (*n* = 72 in SRTR group, Supplementary Table 1). In 72 cases (from 1988 to 2016) of LPTx in SRTR), with some data missing, 72.2% were cluster implantation, and 12.5% techniques involved separated OLT and pancreas transplantation; 40.3% were anastomosed allograft portal to recipient portal vein, and 48.6% were done using portal to iliac cava venous anastomosis. Furthermore, 52.8% operations were completed using a Roux-en-Y fashion for enteric drainage, and 9.7% were bladder drainage. In FAH, all 31 cases were unified using a cluster implantation, portal-to-portal side to end anastomosis, and exocrine enteric drainage. In SRTR group (72 patients from 1988 to 2016), no difference in 1-year patient survival in vascular or exocrine drainage management (60 vs. 59, 66 vs. 57%, Supplementary Table 2).

### Patient and Graft Survival

**Figure 1:**
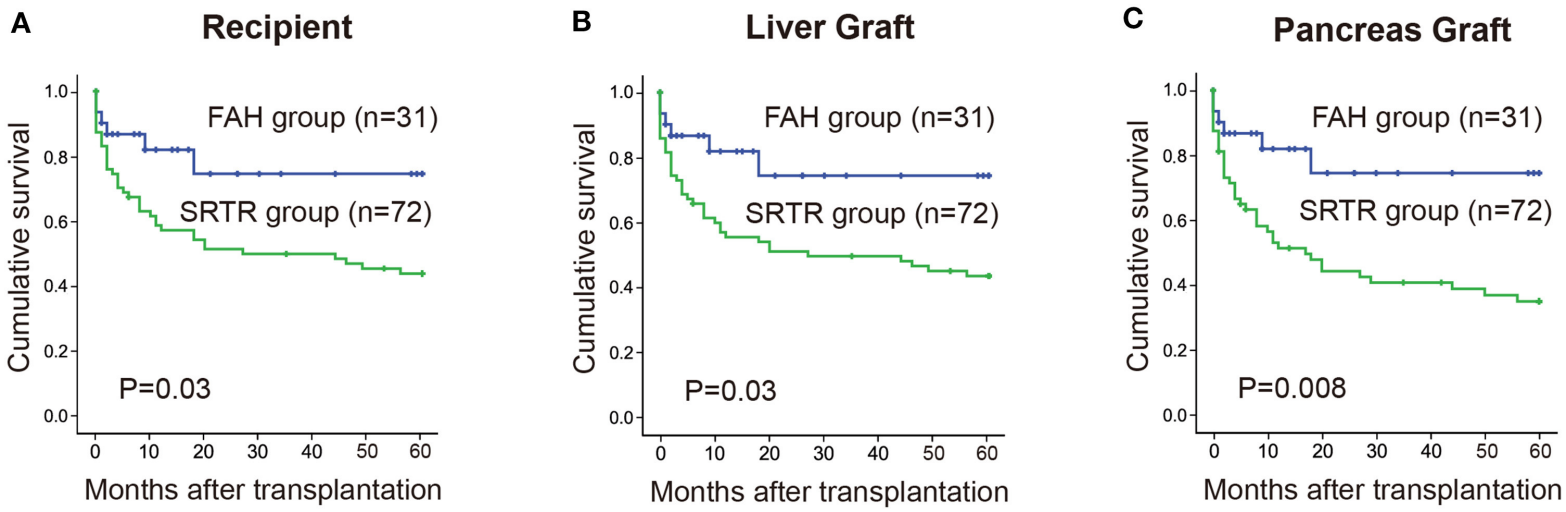
Cumulative survival rate between the FAH group and the SRTR group were analyzed by Kaplan-Meier method (log rank). **(A)** 5-year cumulative survival rate of recipients between two groups. **(B)** 5-year cumulative survival rate of liver graft between two groups. **(C)** 5-year cumulative survival rate of pancreas graft between two groups.

The 1-, 3-, and 5-year cumulative patient survival rates were 81, 74, and 74% in FAH group, respectively, and 51, 47, and 37% in SRTR group, respectively (*P* = 0.023) (Figure 1A). In FAH group, one recipient died of sepsis and multiple organ failure secondary to graft versus host disease on post-operation day (POD) 45. One patient died of sepsis followed by severe pancreatitis on POD 38. Another recipient died of severe pneumonia due to the side effects of chemotherapy against acute mononuclear leukemia at postoperative month (POM) 13. Two patients died from tumor recurrence at POM 18 and 26, respectively. The 1-, 3-, and 5-year cumulative liver graft survival rates were 81, 74, and 74% in FAH group, respectively, and 51, 47, and 37% in the SRTR group, respectively (*P* = 0.023) (Figure 1B). The 1-, 3-, and 5-year cumulative pancreas graft survival rates were 81, 74, and 74% in the FAH group, respectively, and 43, 37, and 25% in the SRTR group, respectively (*P* = 0.004) (Figure 1C). We also compared the survival of the FAH group and the cases in SRTR group from 1988 to 2016 (*n* = 72). The 1-, 3-, and 5-year patient survival, liver graft survival, pancreas graft survival rates in SRTR (*n* = 72) were significantly lower than those of the FAH group (Supplementary Figure 1 and Supplementary Table 3).

### Diabetes During Follow-Up

During follow-up, no diabetes recurrence (0/31) was observed in the FAH group, while the proportion of post-transplant diabetes was 22.2% (4/18) in the SRTR group (*P* = 0.03) (Table 2). In the four recipients in the SRTR with post-transplant diabetes, three were given a diagnosis of T1DM before transplantation. Among them, one recipient took insulin after pancreas graft lost on POM 49 and died on POM 50. The other two recipients' pancreas graft lost function on POM 83 and 84, respectively, and both required insulin therapy. Also, one recipient's pretransplant pancreas diagnosis was unknown, whose pancreas graft died on POM 35. Unlike the former three patients, the other recipient was insulin-independent, and the patient's blood glucose was controlled by dietary measures at last overall follow-up on POM 35.

**Table 2:** Follow-up between two groups.

	**FAH**		**SRTR**		***P***
	***n***	**Proportion**	***n***	**Proportion**	
Diabetes recurrence	0/31	0.0%	4/18	22.2%	0.03
Pancreas acute rejection	0/31	0.0%	6/17	35.3%	<0.001
Liver acute rejection	1/31	3.2%	4/18	22.2%	0.001
Pancreatitis	1/31	3.2%	1/18	5.6%	0.69
Anastomosis leak	2/31	6.5%	0/18	0.0%	0.17
Post-transplant Malignancy	2/31	6.5%	2/18	11.1%	0.97
**IMMUNOSUPPRESSIVE PROTOCOL**					
Maintenance	31/31	100%	5/5	96.6%	1.00
Induction	31/31	100%	1/5	20.0%	<0.001
Ant-rejection	1/31	3.2%	0/5	0.0%	1.00

*FAH, the First Affiliated Hospital, Sun Yat-sen University; SRTR, Scientific Registry of Transplant Recipients*.

### Complication Profiles

The incidence of postoperative complications is summarized in Table 2. The data during follow-up were missing in varying degrees. Six of 17 (35.3%) recipients experienced pancreas acute rejection in the SRTR group, while no recipients in the FAH group did (*P* < 0.001). One of 18 (5.6%) recipients in the SRTR group and 1/31(3.2%) recipients in the FAH group were diagnosed with pancreatitis during the follow-up (*P* = 0.69). Four of 18 (22.2%) recipients suffered from liver acute rejection in the SRTR group, while 1/31 (3.2%) recipients in the FAH group did (*P* = 0.001). No difference was observed in anastomosis leak. Also, malignancy recurred in 2/18 (11.1%) recipients in the SRTR group and 2/31(6.5%) in the FAH group; however, the difference was not significant (*P* = 0.97).

## Discussion

In the 1990s, reports of “multi-organ” or “cluster” transplantations usually included the liver and pancreas. The classical multivisceral transplantation (MVT) should firstly resect abdominal organs (liver, pancreas, intestine, and stomach), then implant the cluster allograft (liver, pancreas, and intestine), which is a milestone in the history of transplantation. Since its inception, LPTx was included in upper-abdominal MVT with an inferior outcome. However, during the development of transplantation, LPTx seems to not be the priority in patients with liver and pancreas failure (13, 14). The annual number of LPTx performed in the United States and worldwide is relatively sporadic. In the last decade, only 12 cases were recorded in the SRTR database. Using an available database with patients undergoing LPTx in the United States and our single-center cases, we assessed the indications, surgical techniques, and outcomes of LPTx recipients.

The indications of LPTx altered over the past decades. Candidates for this procedure generally had a condition that was inappropriate for standard medical or surgical therapy and considered terminal or sometimes contraindicated for surgery. The three major indications of LPTx in the SRTR database are cirrhosis (including autoimmune, HCV, and HBV), abdominal malignant tumor, and CF. Only six reported cases of LPTx exist in patients suffering from liver cirrhosis and DM before 2000, according to the summary by Trotter et al., with varying degrees of success, which they blamed on the rarity of simultaneous occurrence of the two diseases (13). However, up to 19.6% of liver transplant candidates have concurrent T2DM in the SRTR database, which maintains a steady growth from 2003 to 2015. Patients with ESLD and DM often undergo an OLT and insulin therapy (2, 3). In some patients, glucose control is impaired even after OLT due to the use of immunosuppressive agents (15). It is well-demonstrated that preexisting and new-onset DM elevate the risk of mortality and complications in liver transplant recipients (7). A study of 85,000 liver transplant recipients using SRTR database reveals that 11.2% of recipients have pretransplant DM, and ~10% post-operation deaths are associated with pretransplant DM (4). Furthermore, DM is reported to increase the risk of HCC in the presence of other risk factors, such as hepatitis C or B or alcoholic cirrhosis (16). Notably, new-onset DM after OLT affects not only graft function, but also cardiovascular disease morbidity and mortality (17). Therefore, LPTx might provide a possible best treatment of choice for patients with ESLD and concurrent DM.

In 2009, Kornberg et al. reported LPTx in 14 patients with liver cirrhosis and insulin- dependent T2DM (18). In their study, all patients were rendered independent from insulin therapy shortly after transplantation. The recurrence rate of exogenous insulin dependence was low (1/14), and the 7-year patient survival rate was as high as 64.2%. The study has proven that LPTx is technically feasible and able to achieve an excellent long-term control of glucose metabolism in this group of patients. Nevertheless, in Kornberg et al.'s study, most patients had normal pre-transplant C-peptide and insulin values, indicating relative insulin deficiency or insulin resistance. In our previous study, we compared the outcome between 23 patients who received SMT and 21 patients who received OLT (11). The study provides evidence that in patients with T2DM and severely impaired pancreatic cell function, SMT can restore control of glucose metabolism and offers excellent quality of life. In the current study, all 31 patients were diagnosed with T2DM long before transplantation, and severe insulin deficiency developed when they were admitted to our transplant center. No DM reoccurred during follow-up, while the DM recurrent rate was 22.2% in the SRTR group. However, it is still unclear which group of T2DM patients would benefit most from this procedure.

Whether LPTx is performed using a cluster technique or a non-cluster technique is still an issue of debate. In 72 cases of LPTx in SRTR, 26.8% of the cases were performed using non-cluster technique, which means a standard orthotropic OLT followed by heterotopic placement of the pancreas allograft. In the SRTR group, the patient survival rates are comparable between patients undergoing the cluster and non-cluster procedure (53vs. 72%, *P* = 0.26). Moreover, we observed no difference in 1-year patient survival in vascular or exocrine drainage management (60 vs. 59%, 66 vs. 57%, Supplementary Table 2). Although outcomes are comparable with different transplant techniques in the SRTR group, the modified cluster procedure SMT in FAH group provides a superior outcome. The surgical technique en-bloc cluster implantation with liver and pancreas grafts originates from MVT described by He et al. (11) and Starzl et al. (19). As opposed to classical MVT, patients with ESLD and DM in SMT group usually need resection of the diseased liver but not the diseased pancreas (14). Firstly, we modified the hepatic artery anastomosis using the interposition artery reconstruction in advance at the back table and avoided clamping the aorta. Secondly, side-to-side duodeno-jejunal anastomosis was done to ensure the exocrine pancreatic drainage (11). Thus, only three vascular anastomoses and one digestive tract anastomosis needed to be done, obviating the biliary anastomosis and another incision. Therefore, compared to the separated implantation of liver and pancreas, the major advantage of SMT technique is its simplicity. In addition, the pancreas graft is placed in the physiologic position, ensuring the intact blood supply and the natural venous drainage into the donor liver. Better metabolic control is observed in the pancreatic portal drainage technique. Furthermore, upper endoscopy is considered to be easy access to predict pancreas rejection through duodenum mucosa biopsy (18). Henn C et al. indicated that pancreas-related complications, such as graft pancreatitis or pancreatic vessel thrombosis, may compromise liver grafts (20). In FAH case series, one patient suffered from severe pancreatitis which subsequently resulted in sepsis, and finally died of multiple organ failure.

Because the pancreas allograft is more immunogenic than the liver, addition of a pancreas to a liver graft may increase the risk of acute rejection (9). On the other hand, it has been reported that liver allograft can induce tolerance to other organs from the same donor. In a more recent study, the disparity in rejection rates (50 and 17%) between the modified MVT (without the liver) and MVT groups highlights the importance of donor liver in attenuating rejection risk (21). Considering the complexity of the immune response in SMT, an effective but not overdose immunosuppressive regimen similar is required. This application is critical to decrease complications, such as infections. Therefore, a minimized immunosuppressive protocol was introduced in the SMT recipients in our center, which proved to be a safe and effective regimen with low incidences of both rejection and infection. In the analysis, the incidence of pancreas graft acute rejection rate was significantly higher in SRTR group than that in FAH group. However, we were not able to compare the protocols because the data concerning immunosuppressant doses were largely missing in the SRTR database.

Owing to the development of a voluntary organ donation system in China (22), we have witnessed a steady increase in SMT volume. We have performed 31 cases of SMT, while the largest single-center experience of LPTx is 28 in those 72 cases from SRTR. A principle limitation of this study is the incomplete data recorded in LPTx in the SRTR database, which may result in a significant bias when comparing indications and complications. Furthermore, the follow-up in SRTR started earlier than in the FAH group, but only 10 cases of LPTx were recorded in the last decade. Therefore, the outcomes between the two groups are not well-compared. So we compared patients in similar period in both group. But overall, there were still bias including baseline characteristics, indications, and peri-operation management. The number of cases made it difficult to compare the techniques. We recognize that the number of SMT or LPTx is still small worldwide. In this study, we offer the SMT procedure for ESLD and T2DM patients. A prospective, controlled, and multicenter study is required to confirm the long-term outcome of SMT or LPTx.

In conclusion, our study compares the indication, technical difference, and outcome of combined liver and pancreas transplantation between the SRTR database and a single center in China. We review the development tendency of LPTx in the United States and describe that SMT, also known as simultaneous en-bloc LPTx, can provide a safe and effective option for patients with ESLD and concurrent T2DM. This surgical procedure has become a conventional transplantation technique for liver transplantation in our center and may expanded the surgical indications shown in SRTR. Moreover, it represents the development and maturity of a novel program with improving outcomes related to increased experience and volume. Further modification of this technique and more precise definition of the indication would greatly benefit this large group of transplant candidates.

## Data Availability Statement

All datasets generated for this study are included in the article/Supplementary Material.

## Ethics Statement

The studies involving human participants were reviewed and approved by the Ethical Committee at the First Affiliated Hospital of Sun Yat-sen University. The patients/participants provided their written informed consent to participate in this study.

## Author Contributions

CL: conceptualization, project administration, data curation, formal analysis, and writing-original draft. WZ: project administration, formal analysis, and writing-original draft. MY: investigation, methodology, and writing-editing. QZ: project administration, data curation, and formal analysis. WJ: data curation, formal analysis, and investigation. LW: data curation and investigation. YM, AH, GW, and XZ: data curation and formal analysis. ZG: funding acquisition, project administration, and writing-review. DW and XH: funding acquisition, project administration, writing-review, and supervision. All authors: contributed to the article and approved the submitted version.

## Conflict of Interest

The authors declare that the research was conducted in the absence of any commercial or financial relationships that could be construed as a potential conflict of interest.

## Abbreviations

LPTx: combined liver and pancreas transplantation
SRTR: Scientific Registry of Transplant Recipients
FAH: the First Affiliated Hospital, Sun Yat-sen University
SMT: simplified multivisceral transplantation
DM: diabetes mellitus
HBV: hepatitis B virus
HCV: hepatitis C virus
HCC: hepatocellular carcinoma
T2DM: type 2 diabetes mellitus
T1DM: type 1 diabetes mellitus
CF: cystic fibrosis
ESLD: end-stage liver diseases
OLT: orthotopic liver transplantation
OPTN: Organ Procurement and Transplantation Network
HRSA: The Health Resources and Services Administration
BMI: body mass index
MELD: model of end-stage liver disease
MVT: multivisceral transplantation
MMF: mycophenolate mofetil
DBD: donation after brain death
DCD: donation after cardiac death
POD: post-operative day
POM: postoperative month
PNF: primary non-function
GVHD: graft versus host disease
AML: acute myeloid leukemia.
