# Supplementary material for: Outcomes of Combined Liver and Pancreas Transplantation: A Review of the SRTR National Database and a Report of the Largest Single Center Series
Source: Front Med (Lausanne). 2020 Oct 19;7:542905. doi: 10.3389/fmed.2020.542905 (PMC7605456; doi:10.3389/fmed.2020.542905)
Supplement: Supplementary file 1 [file Data_Sheet_1.docx]

Supplement Table 1. comparison of surgical techniques between the FAH group and the SRTR group

|  | FAH | SRTR | P |
| --- | --- | --- | --- |
| Implantation technique |  |  | 0.001 |
| Cluster | 31（100%） | 52（72.2%） |  |
| Non-cluster | 0（0%） | 19（12.5%） |  |
| Venous Vascular Management |  |  | 0.001 |
| Systemic system (iliac: cava) | 0（0%） | 35（48.6%） |  |
| Portal system (portal or tributaries) | 31（100%） | 29（40.3%） |  |
| Exocrine drainage |  |  | 0.04 |
| Enteric drainage (Roux-en-y) | 31（0%） | 38（52.8%） |  |
| Bladder drainage | 0（100%） | 7（9.7%） |  |

Abbreviations: FAH, the First Affiliated Hospital, Sun Yat-sen University; SRTR, Scientific Registry of Transplant Recipients

| Implantation technique | Cluster | Non-cluster |  |
| --- | --- | --- | --- |
|  | 53% | 72% | P=0.26 |
| Venous vascular management | Systemic system (iliac: cava) | Portal system (portal or tributaries) |  |
|  | 60% | 59% | P=0.91 |
| Exocrine drainage | Enteric drainage (Roux-en-y) | Bladder drainage |  |
|  | 66% | 57% | P=0.54 |

Supplement Table 2. 1-year cumulative survival rate among recipients in the SRTR group, by surgical techniques

Abbreviations: SRTR, Scientific Registry of Transplant Recipients

Supplementary Table 3. Summary of patient and donor characteristics.

|  | FAH (n=31) | SRTR (n=72) | *P* |
| --- | --- | --- | --- |
| Recipient characteristics |  |  |  |
| Gender (male) | 30/31 | 45/72 | 0.001 |
| Age (years) | 56.1±9.8 (33-73) | 40.2±12.5 (19-66) | 0.001 |
| BMI (kg/m^2^) | 23.7±3.6 (18.8-32.1) | 23.4±4.8 (16.24-34.9) | 0.73 |
| MELD score | 19.0±12.3 (6-49) | 22.6±7.6 (6-40) | 0.19 |
| Follow up (months) | 13(2,28) (0-78) | 11(2,83.5) (0-323) | 0.57 |
| Hospital stays (days) | 37(26,47) (10-138) | 26(13,53) (0-148) | 0.10 |
| Diagnosis |  |  |  |
| Pancreas diagnosis |  |  | <0.001 |
| Diabetes mellitus-type I | 0/31 | 27/43 |  |
| Diabetes mellitus-type II | 31/31 | 6/43 |  |
| Other (pancreatic cancer, cystic fibrosis, etc.) | 0/31 | 10/43 |  |
| Liver diagnosis |  |  | <0.001 |
| HCC | 18/31 | 5/72 |  |
| Cirrhosis | 11/31 | 18/72 |  |
| Cholangiocarcinoma | 1/31 | 11/72 |  |
| Malignancy | 0/31 | 16/72 |  |
| Other | 1/31 | 22/72 |  |
| Hepatitis virus |  |  |  |
| HBV N/P | 1/30 | 65/4 | 0.96 |
| HCV N/P | 31/0 | 29/2 | 0.49 |
| Donor characteristic |  |  |  |
| Gender (male) | 24/31 | 50/72 | 0.13 |
| Age (years) | 29.0±11.8 (10-56) | 25.2±9.9 (8-46) | 0.10 |
| BMI (kg/m^2^) | 21.4±2.8 (17.0-26.7) | 22.1±4.2 (9.5-35.4) | 0.53 |
| Donation category |  |  | <0.001 |
| DBD | 13 | 33 |  |
| DCD | 17 | 0 |  |
| DBCD | 1 | 0 |  |
| Cold Ischemia Time (hour) | 7.7±1.3 (6.0-11.0) | 9.6±5.1 (3.0-35.3) | 0.006 |

Abbreviations: FAH, the First Affiliated Hospital, Sun Yat-sen University; SRTR, Scientific Registry of Transplant Recipients; BMI, body mass index; MELD, model for end-stage liver disease; HCC, hepatocellular carcinoma; HBV, hepatitis B virus; HCV, hepatitis C virus; DBD, donation after brain death; DCD, donation after cardiac death. Estimates of variance are standard deviations.

**Supplementary Figure 1**. Cumulative survival rate between the FAH group (n=31) and the SRTR (n=72) group were analyzed. (A) 5-year cumulative survival rate of recipients between two groups. (B) 5-year cumulative survival rate of liver graft between two groups. (C) 5-year cumulative survival rate of pancreas graft between two groups.
