# Supplementary figures and images for: Outcomes of Combined Liver and Pancreas Transplantation: A Review of the SRTR National Database and a Report of the Largest Single Center Series
Source: Front Med (Lausanne). 2020 Oct 19;7:542905. doi: 10.3389/fmed.2020.542905 (PMC7605456; doi:10.3389/fmed.2020.542905)

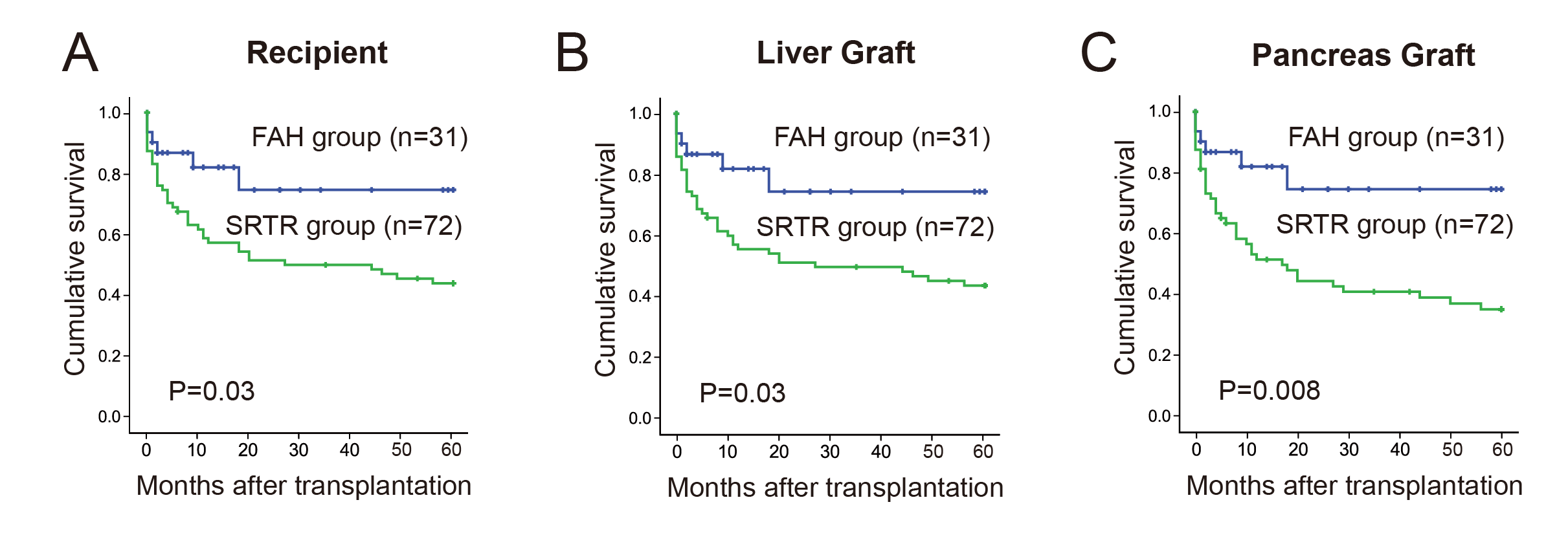

Supplement: Supplementary file 2 [file Image_1.tif]
